# Sex-specific cytokine signatures as predictors of anti-PD1 therapy response in non-small cell lung cancer

**DOI:** 10.3389/fimmu.2025.1583421

**Published:** 2025-06-16

**Authors:** Catherine Taylor, Ammar Sabir Cheema, Karama Asleh, Nicholas Finn, Mahmoud Abdelsalam, Rodney J. Ouellette

**Affiliations:** ^1^ Atlantic Cancer Research Institute, Moncton, NB, Canada; ^2^ Department of Pathology and Laboratory Medicine, Dalhousie University, Halifax, NS, Canada; ^3^ Dr Léon-Richard Oncology Center, Vitalité Health Network, Moncton, NB, Canada; ^4^ Division of Medical Oncology, Moncton Hospital, Moncton, NB, Canada; ^5^ Department of Chemistry & Biochemistry, Université de Moncton, Moncton, NB, Canada

**Keywords:** NSCLC, immune checkpoint inhibitor, cytokine, chemokine, CXCL12, CXCL10, sex disparity

## Abstract

**Background:**

The introduction of immune checkpoint inhibitors (ICI) as first-line therapy in the treatment of non-small cell lung cancer has dramatically improved response rates. However, more than half of NSCLC patients receiving ICI fail to have a durable response to treatment and therefore the identification of circulating biomarkers to improve patient stratification is required. Cytokines and chemokines are critical mediators of immune responses, affecting tumor progression and immune evasion mechanisms. Thus, profiling circulating cytokines is particularly important, as these signaling molecules may provide valuable insights into predicting response and resistance to ICI.

**Methods:**

Twenty-four circulating chemokines and cytokines were profiled in NSCLC patient plasma collected either prior to treatment or while on-treatment with anti-PD1 therapy and correlated to treatment response as well as to progression-free survival (PFS) and overall survival (OS). Sex-disparities in correlations of cytokines to response and survival was analyzed.

**Results:**

Regardless of sex, baseline levels of CCL5/RANTES were associated with anti-PD1 treatment response, while CXCL5 was associated with response in males and CXCL10 was elevated in female responders to anti-PD1 treatment. VEGF and CD40L were associated with short PFS and OS, while CCL5 and CXCL5 were correlated to longer PFS and OS. Sex disparities in baseline cytokine levels were also observed. CCL5 was significantly correlated to PFS and OS in females but not males, and CXCL10 was found to be predictive of longer OS in females only. VEGF was found to be a better predictor of response t to anti-PD1 in females, while CXCL12 was found to be associated with short PFS and OS in males but not females. Uniform Manifold Approximation and Projection (UMAP) dimension reduction method and k-means clustering analysis identified a cluster of male patients with short PFS characterized by elevated baseline levels of VEGF, CCL4, CCL5, CCL20, and CXCL2.

**Conclusions:**

Plasma cytokine levels can be useful biomarkers for predicting response to anti-PD1 therapy in NSCLC patients. However, the data presented in this study demonstrate that sex needs to be considered as an important variable in biomarker studies in immuno-oncology due to sex disparities in correlations of cytokines to anti-PD1 treatment response.

## 1 Introduction

In the rapidly developing field of precision medicine, the use of liquid biopsy to improve patient stratification and optimize treatment of patients receiving immuno-oncology (I-O) therapies is of paramount importance. The use of immune checkpoint inhibitors (ICIs) targeting programmed cell death protein 1 (PD1) and its ligand PDL1, have dramatically improved the treatment landscape for non-small cell lung cancer (NSCLC) patients. However, approximately 50% of patients fail to derive a significant clinical benefit from ICI treatment due to either primary or acquired treatment resistance (1). Currently, the only clinical biomarker driving treatment selection in practice is PDL1 tumor proportion score (TPS), which is not a reliable predictor of ICI response and survival benefit (2, 3).

ICIs are designed to inhibit immune checkpoint proteins expressed by tumor cells to prevent tumor evasion of T cell-mediated immune surveillance. First-line treatment with the anti-PD1 immune checkpoint inhibitor pembrolizumab is currently standard of care for patients with metastatic NSCLC who are not candidates for surgical resection or targeted therapies (4). Pembrolizumab is typically given as a monotherapy for patients with tumors expressing PDL1 or in combination with chemotherapy for patients with tumors with low PDL1 expression. Although durable responses are observed in many patients, there remain a large fraction of patients who fail to benefit from this strategy (1). The presence of anti-tumor immune activation, in the form of antigen presentation as well as activation and infiltration of effector T cells into the tumor microenvironment (TME) is generally considered to be necessary for a robust response to ICI therapy (5). However, due to the difficulties of measuring intra-tumoral biomarkers, research has switched focus to using liquid biopsy to identify immune-related biomarkers that are predictive of ICI response. To maximize the potential of ICI therapy in the treatment of lung cancer, it is therefore crucial to be able to not only develop robust biomarkers to identify patients who will respond to the therapy but also to monitor the onset of acquired resistance and improve our understanding of the mechanisms underlying treatment failure.

The sex of patients receiving I-O for non-reproductive cancers is not currently given sufficient weight in standard of care, despite well described sex-related differences in immune function (6). Sex and aging have profound effects on the composition of circulating immune cells (7). Sex-related differences in immune responses to both pathogens and self-antigens is well described, with females typically exhibiting stronger innate and adaptive immune responses (8, 9). This is highlighted by the more robust immune response to vaccination and infections observed in women (7), but also by the increased incidence of inflammatory and auto-immune diseases (9). A better understanding of the differences in patterns of innate immune responses in the tumor microenvironment between male and female cancer patients is critical for understanding sex-related differences in disease progression and immunotherapy treatment response. Although lung cancer is not among the cancers traditionally thought of as being hormone sensitive, both male (androgens, e.g. testosterone) and female (e.g. estrogen) sex hormones have been reported to influence the pathophysiology of lung cancer (10, 11) and lung cancer cells have been reported to produce estrogen through the action of aromatase activity (12). Most lung cancers are responsive to estrogen signaling due to the expression of estrogen receptors (ERs), which contribute to cancer progression by promoting proliferation, migration, and angiogenesis (13).

The use of immune checkpoint inhibitors has dramatically improved survival rates for patients with advanced cancers, but sex-related differences in the degree of benefit have been observed with females deriving more benefit from immunotherapy than males (14). The presence of infiltrating CD8+ T cells in the TME is a known predictor of anti-PD1 response (5) and therefore profiling circulating cytokines and chemokines has gained interest to identify potential biomarkers of response. Although a few studies have investigated the use of plasma cytokines as predictors of response to immunotherapy in NSCLC patients (15–17), these studies generally did not include sex as a variable in their analysis. The purpose of the current study was to profile 24 inflammatory and immune-related plasma cytokines at baseline in immunotherapy-naïve NSCLC patients receiving anti-PD1 treatment and to evaluate those with potential as predictive and prognostic biomarkers that correlate to treatment response and progression-free survival (PFS). A secondary aim was to investigate sex-related discrepancies in the predictive behavior of baseline cytokines. On-treatment changes in cytokines were also analyzed in a subset of patients.

## 2 Materials & methods

### 2.1 Patient cohort

Plasma samples for a cohort of 55 consented patients with metastatic NSCLC treated with anti-PD1 therapy between September 2018 and May 2024 were obtained from the CHU Dumont Biobank. Plasma collected prior to the initiation of treatment was included in the study, as well as post-treatment samples when available. The plasma samples were collected and analyzed under IRB-approved protocols and studies were conducted in accordance with the Declaration of Helsinki and with approval from the research ethics board of the Vitalité Health Network. All patients received treatment with at least two cycles of pembrolizumab. Patients who experienced disease progression within 6 months or less of the initiation of treatment with pembrolizumab were classified as ‘non-responders’ to treatment. Responders were defined as patients who exhibited a clinical response with either stable disease or reduced tumor volume, as determined by imaging, lasting for more than 6 months. PFS was defined as the duration in days between the date of the first treatment dose and the date of either disease progression or death due to any cause. Overall survival (OS) was defined as the duration in days between the date of the first treatment dose and death due to any cause.

### 2.2 Cytokine and chemokine profiling

The levels of circulating cytokines and chemokines in plasma was measured using a 22-plex Luminex Human Cytokine Discovery Kit (Biotechne, Minneapolis, MN, USA) which was used to quantify CCL2, CCL4, CCL7, CCL20, CCL22, CXCL1, CXCL2, CXCL5, CXCL10, CXCL11, GM-CSF, IL-6, IL-8, IL-10, IL-15, IL-23, G-CSF, TRAIL, TNF-α, CD40L, PDL1, and VEGF). CCL5 was analyzed using a Luminex single-plex assay (Biotechne, Minneapolis, MN, USA). The Luminex assays were performed according to the manufacturer’s instructions and analyzed using a Bio-Plex 200 System and Bio-Plex Manager software. CXCL12 was quantified by using a DuoSet™ ELISA kit (R&D Systems, Minneapolis, MN, USA). For values that were below the limit of detection, we substituted the lowest measurable value for that biomarker.

### 2.3 Statistical analysis

Statistical significance was calculated in GraphPad Prism vs 10.0.2 (GraphPad Software, San Diego, CA, USA) using two-tailed t-tests and Mann-Whitney U tests. Kaplan-Meier plot generation and univariate and multivariate cox proportional hazards model analyses were performed in R environment vs 4.4.0 (R Studio, Boston, MA, USA) using survminer, survival, ggplot2, and readr packages. The optimal cutpoint value in bootstrap samples for each biomarker was determined using the best Cox model fit internally validated on 500 bootstrap samples using the survival package. In instances where the optimal cutpoint was not appropriate, the median cutpoint was used. Pearson correlation analysis was performed to assess correlations between baseline biomarker levels. The correlation matrix and significance levels were visualized using the ‘corrplot’ function from the corrplot package (version 0.92) and insignificant correlations (p<0.05) based on computed p-values were excluded.

Clustering and Uniform Manifold Approximation and Projection (UMAP) analysis were conducted using R version 4.3.2. Prior to UMAP analysis, the data was normalized by mean-centering using the scale function with default parameters from base R and cytokines with missing values were removed. Dimensionality reduction using principal component (PC) analysis (PCA) was performed using the ‘PCA’ function from the FactoMineR package (version 2.9). The selection of top contributing principal components was guided by a scree plot, generated using the ‘fviz_eig’ function from the factoextra package (version 1.0.7) and four PC were used, depending on the dataset. Clustering was performed on the reduced data using the ‘Kmeans’ function from base R. The optimal number of clusters was determined using the ‘fviz_nbclust’ function from the FactoExtra package (version 1.0.7). Clusters were generated by running k-means on the selected PC.

## 3 Results

### 3.1 Patient characteristics

**Figure 1:**
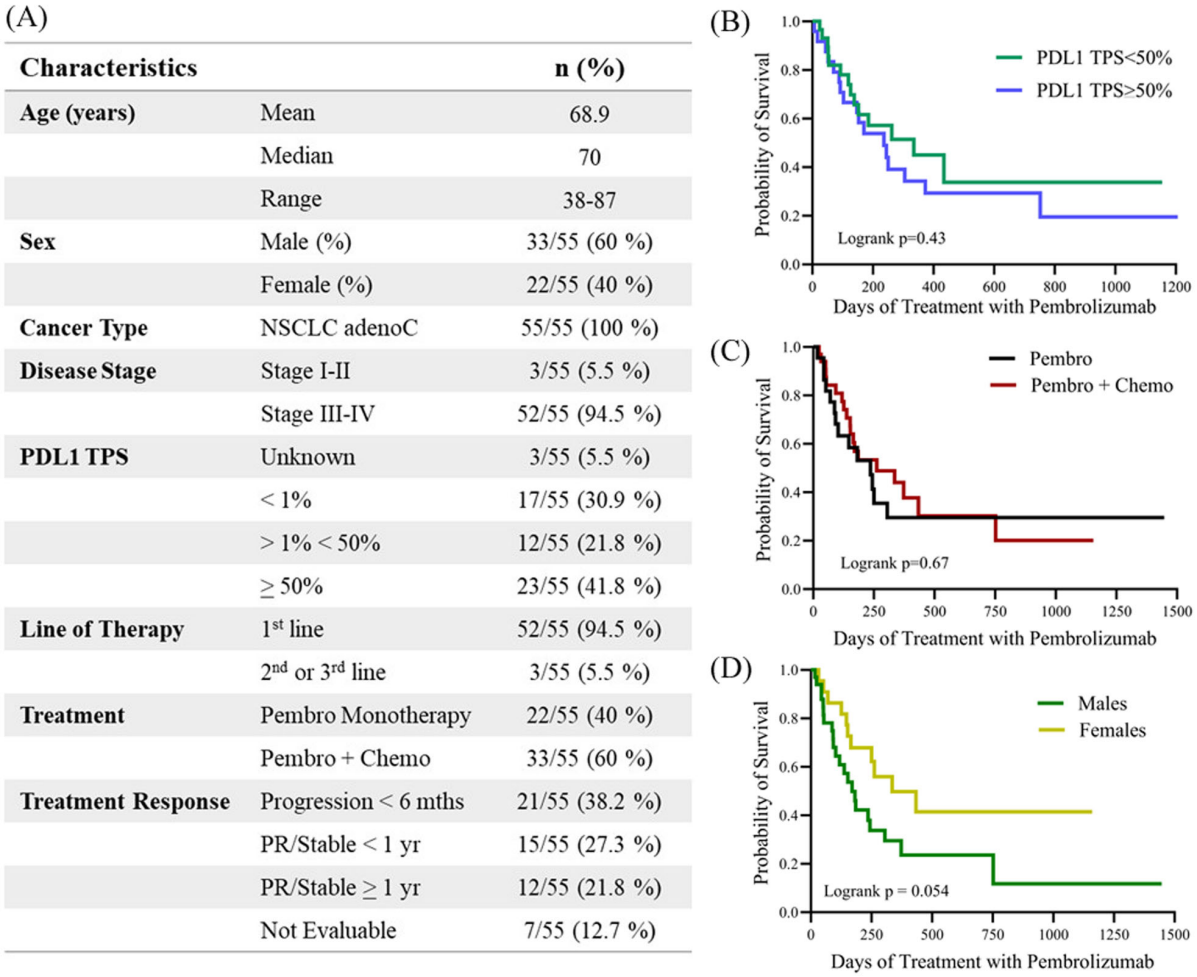
**(A)** Table describing the clinicopathological characteristics of the study. Kaplan-meier plots demonstrating the correlation of PFS with clinicopathological characteristics including **(B)** PDL1 tumor proportion score (TPS) <50% versus >50, **(C)** treatment with either pembrolizumab monotherapy (Pembro) versus in combination with chemotherapy (Pembro + Chemo), and **(D)** male versus female. Logrank p values are shown. PDL1, Programmed death-ligand 1; TPS, tumor proportion score; PR, partial response.

In this study, soluble cytokines were profiled in EDTA plasma samples collected from 55 patients diagnosed with NSCLC who received treatment with pembrolizumab. Patient baseline characteristics (**Figure 1**) were representative of a population not previously treated with ICIs and who received pembrolizumab primarily as first-line treatment (94.5% of patients). The study included thirty-three (60%) male and twenty-two (40%) female patients. The PFS for all patients in the study was 244 days, while the median PFS of female patients was 335 days compared to 170 days for males (HR for males= 2.02; 95% CI 0.97-4.18; p=0.054). Sex was found to be a predictor of OS in this study with males exhibiting worse outcomes than females (**Supplementary Figure S1**). The median OS for the total cohort was 479 days, respectively, while median OS was 670 days for females compared to 375 days for males (HR for males = 2.26; 95% CI = 1.03-4.93, p=0.036). The median age for the entire 55 patient cohort was 70 years, with the median age of the males in the cohort being 71 years compared to 68.5 years for the female population. The median OS of patients <70 years old was 670 days compared to 468 days for patients >70 years old (HR=1.36; 95% CI 0.67-2.77; p= 0.39).

PDL1 TPS was available for 52 of the 55 patients (95%), with PDL1 ≥ 50% reported in 23 (41.8%) patients for whom data was available. PDL1 TPS >1% and <50% was reported in 12 patients (21.8%) and PDL1 <1% in 17 (30%) patients. In this study we did not observe any differences in either PFS or OS associated with PDL1 TPS status. Patients with PDL1 TPS<50% had a mean PFS of 335 days compared to 244 days for patients with PDL1 TPS>50% (HR for PDL1 TPS>50% = 1.24, 95% CI 0.61-2.56; p=0.5). Similarly, patients with low PDL1 (TPS<50%) survived for an average of 479 days compared to 468 days for patients with PDL1 TPS>50% (HR for PDL1 TPS>50% = 1.08, 95% CI 0.51-2.28; p=0.8). Twenty-two (40%) of the patients were treated with pembrolizumab monotherapy while thirty-three (60%) of the patients received pembrolizumab in combination with chemotherapy. The median PFS for patients receiving monotherapy was 236 days compared to 262 days for patients also receiving chemotherapy (HR for combination therapy = 0.90; 95% CI 0.45-1.80; p=0.67).

Patients received at least two cycles of pembrolizumab and were followed for a minimum of 6 months. Forty-eight (87.2%) patients were evaluable for response to pembrolizumab treatment. Twenty-seven (49.1%) patients were classified as ‘responders’, while 21 patients (38.2%) progressed on treatment within 6 months and were classified as ‘non-responders’. Seven patients were not evaluable for response to anti-PD1 therapy because they were lost to follow-up or stopped anti-PD1 treatment due to autoimmune-related side effects. The median time of follow-up was 356 days (range 17–1855 days). At the last data cut-off, 16 patients (29.1%) were alive with no evidence of progressive disease, and 6 (10.9%) of them had completed 2 years of anti-PD1 therapy and remained in remission.

### 3.2 Plasma chemokine and cytokine profiling and correlation to I-O response

As the activity of T lymphocytes within the TME is known to be a predictor of response to PD-1 blockade, we evaluated the levels cytokines and chemokines related to T cell activity, including IL-15, GM-CSF, CD40L, PDL1, TRAIL, and TNF-α, as well as cytokines involved with angiogenesis (VEGF), immune cell chemotaxis (CXCL1, CXCL2, CXCL5, CXCL10, CXLC11, CCL2, CCL4, CCL5, CCL7, CCL22, IL-8, G-CSF), macrophage responses (CCL20, CXCL12), and inflammation (IL-6, IL-10, IL-23). The cytokines and chemokines were quantified in patient plasma using Luminex assays, except for CXCL12, which was quantified by ELISA. Of the 24 cytokines measured, 11 cytokines (CCL7, CXCL1, CXCL11, GM-CSF, IL-8, IL-15, TRAIL, G-CSF, IL-10, IL-23, and TNF-α) were detected in <50% of the samples and excluded from the analysis. Thirteen different soluble factors, including CCL2, CCL4, CCL5, CCL20, CCL22, CXCL2, CXCL5, CXCL10, CXCL12, IL-6, CD40L, PDL1, and VEGF were included in the full analysis.

At baseline, prior to the initiation of anti-PD1 treatment, plasma CCL5 was significantly higher in responders (63,756 pg/mL) compared to non-responders (47,011 pg/mL, p = 0.0234). A non-significant trend of elevated levels of CXCL10, 70.6 pg/mL in responders compared to 49.5 pg/mL in non-responders (p=0.06), and CXCL5, 593.3 pg/mL in responders compared to 466.9 pg/mL in non-responders (p=0.09), was also observed (**Table 1**).

**Table 1:** Plasma cytokines were measured in all patients (n=55) prior to initiation of treatment with pembrolizumab.

Cytokine/ Chemokine	Responders (pg/ml)	Non-Responders (pg/ml)	*P*
CCL2	95.5 ± 8.4	105.1 ± 11.6	0.52
CCL4	68.7 ± 10.2	124.2 ± 39.5	0.53
CCL5	62756 ± 5300	47011 ± 6842	0.02*
CCL20	105.7 ± 19.4	249.3 ± 111.2	0.62
CCL22	508.7 ± 57.0	553.6 ± 86.0	0.97
CXCL2	310.9 ± 30.7	288.5 ± 48.3	0.34
CXCL5	593.3 ± 75.7	466.9 ± 84.4	*0.09*
CXCL10	70.6 ± 8.3	49.5 ± 5.7	*0.06*
CXCL12	86.3 ± 5.3	103.0 ± 7.3	0.20
IL-6	4.9 ± 1.0	8.1 ± 3.0	0.97
CD40L	650.2 ± 78.1	708.0 ± 149.6	0.69
PDL1	49.6 ± 5.6	45.5 ± 3.8	>0.99
VEGF	18.5 ± 3.1	25.5 ± 5.2	0.74

Baseline plasma cytokine expression levels in responders compared to non-responders to anti-PD1 therapy is shown. Data shown is means ± SEM and p values (Mann-Whitney test). Significant p values (p<0.05) are indicated in bold, near-significant p values (p<0.1) are in italics. * p<0.05.

**Figure 2:**
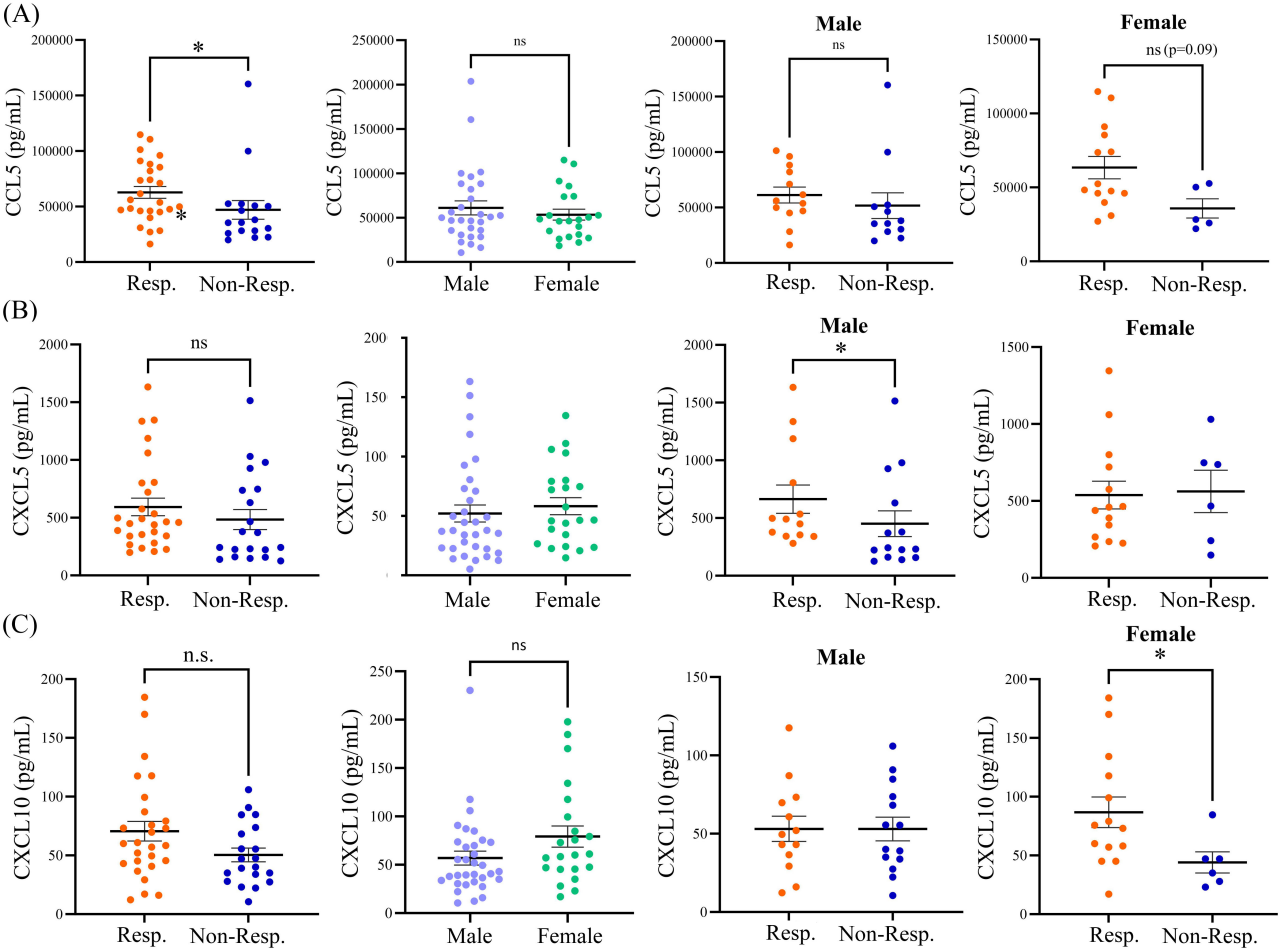
Baseline expression levels of **(A)** CCL5, **(B)** CXCL5, and **(C)** CXCL10 in responders and non-responders from the entire patient cohort, in male versus female patients, in male responders versus male non-responders, and in female responders versus female non-responders. The table shows means ± SEM and p values (Mann-Whitney test; *p<0.05). Resp., responders; Non-Resp., non-responders; n.s., not significant.

The variation of cytokine levels at baseline between males and females (**Table 2**) in the cohort was also explored. The only significant sex-related finding was higher levels of CCL2 in females (134.8 pg/mL) as compared to males (94.6 pg/mL; p=0.017). A non-significant trend of increase in CXCL10 was also observed, with 56.9 pg/mL CXCL10 observed in males compared to 79.2 pg/mL CXCL10 in females (p=0.07) at baseline. We next examined whether there were any differences in the baseline profiles of plasma cytokines between responders and non-responders to anti-PD1 therapy based on sex (**Table 3**). Elevated levels of CCL5 in responders did not reach significance when segregated by sex, suggesting that CCL5 levels associated with treatment response are not influenced by patient sex. We did however observe significantly higher baseline levels of CXCL5 in male responders, with 663.7 pg/mL in male responders compared to 428.9 pg/mL in male non-responders (p=0.03). However, CXCL5 was not associated with response in female patients. In contrast, CXCL10 was found to be significantly correlated to response in females but not males. Baseline levels of CXCL10 were significantly elevated in female responders to anti-PD1 therapy at 87.0 pg/mL compared to 44.2 pg/mL CXCL10 in female non-responders (p=0.05). Therefore, disparities in baseline levels of plasma cytokines CXCL5 and CXCL10 that are correlated to response in a sex-dependent manner was observed (**Figure 2**), suggesting that there may be sex-related differences in the TME of patients who respond to anti-PD1 therapy.

**Table 2:** Baseline plasma cytokine expression levels in male patients compared to female patients.

Cytokine/ Chemokine	Males (pg/ml)	Females (pg/ml)	*P*
CCL2	94.6 ± 9.1	134.8 ± 20.7	0.02*
CCL4	127.5 ± 39.4	93.1 ± 22.2	0.85
CCL5	60887 ± 7885	53332 ± 6138	0.68
CCL20	186.0 ± 65.0	136.9 ± 52.8	0.14
CCL22	536.2 ± 57.4	549.9 ± 75.3	0.97
CXCL2	300.6 ± 34.3	298.4 ± 34.8	0.83
CXCL5	520.8 ± 72.1	582.2 ± 72.1	0.26
CXCL10	56.9 ± 7.1	79.2 ± 11.0	*0.07*
CXCL12	92.7 ± 5.1	91.5 ± 6.8	0.69
IL-6	7.5 ± 1.9	5.0 ± 1.5	0.14
CD40L	652.0 ± 100.3	614.0 ± 92.7	0.99
PDL1	49.8 ± 3.4	46.0 ± 6.5	*0.07*
VEGF	22.7 ± 3.5	20.1 ± 3.8	0.57

The table shows means ± SEM and p values (Mann-Whitney test). Significant p values (p<0.05) are indicated in bold, near-significant p values (p<0.1) are in italics. * p<0.05.

**Table 3:** Baseline plasma cytokine expression levels in male responders compared to male non-responders to anti-PD1 therapy and female responders compared to female non-responders to anti-PD1 therapy.

Cytokine/ Chemokine	Males (pg/ml)		*P*	Females (pg/ml)		*P*
	Responders	Non-Responders		Responders	Non-Responders	
CCL2	86.0 ± 12.7	94.6 ± 16.0	0.65	106.7 ± 10.7	123.4 ± 12.6	0.49
CCL4	57.0 ± 10.2	112.5 ± 48.4	0.64	79.64 ± 17.0	119.8 ± 72.7	0.95
CCL5	61260 ± 7103	51671 ± 11598	0.14	63408 ± 7582	35828 ± 6462	*0.09*
CCL20	138.0 ± 32.0	249.8 ± 140.8	0.78	75.7 ± 20.7	248.0 ± 185.0	0.55
CCL22	568.7 ± 102.7	488.5 ± 80.0	0.62	452.9 ± 54.6	716.5 ± 215.5	0.60
CXCL2	283.8 ± 41.2	310.1 ± 62.9	0.79	336.1 ± 45.7	234.2 ± 64.5	0.24
CXCL5	663.7 ± 122.4	428.9 ± 105.7	0.03*	538.2 ± 90.3	562.0 ± 137.6	0.78
CXCL10	53.1 ± 8.1	51.5 ± 7.2	0.81	87.0 ± 13.0	44.2 ± 9.0	0.05*
CXCL12	81.7 ± 8.7	102.9 ± 7.5	0.10	90.6 ± 6.4	103.3 ± 19.2	>0.99
IL-6	5.5 ± 1.5	8.84 ± 3.9	0.72	4.3 ± 1.4	6.26 ± 4.3	0.89
CD40L	646.8 ± 93.2	758.7 ± 199.1	0.78	653.43 ± 126.8	581.0 ± 177.1	0.21
PDL1	49.2 ± 5.8	47.1 ± 4.7	0.79	50.0 ± 9.7	41.4 ± 6.8	*0.07*
VEGF	16.8 ± 3.7	27.0 ± 6.9	0.72	20.1 ± 5.0	21.7 ± 8.3	0.78

The table shows means ± SEM and p values (Mann-Whitney test). Significant p values (p<0.05) are indicated in bold, near-significant p values (p<0.1) are in italics. * p<0.05.

### 3.3 Changes in cytokine profiles post-treatment

**Figure 3:**
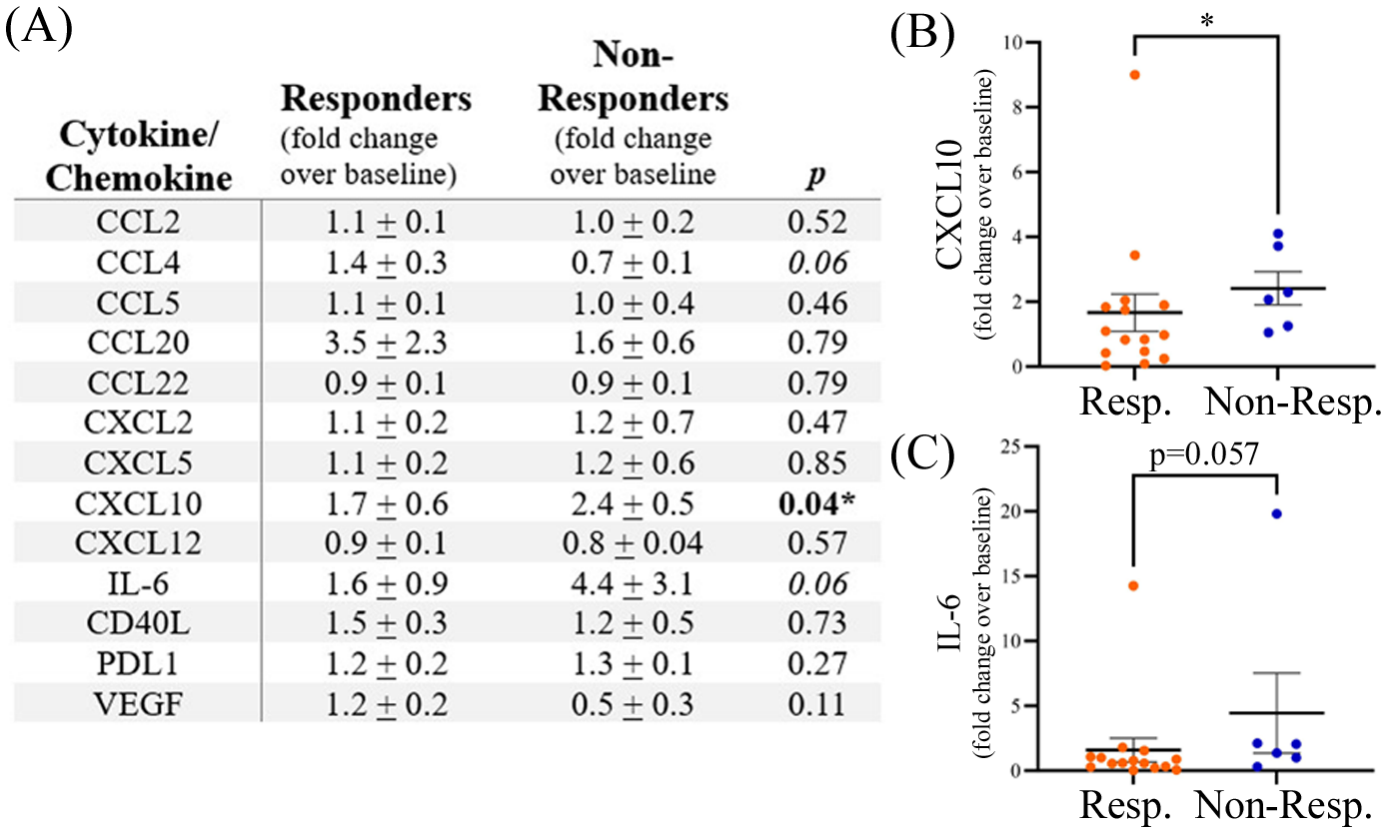
**(A)** Expression of plasma cytokines post-treatment with anti-PD1 therapy shown as fold change over baseline. The table shows means ± SEM and p values (Mann-Whitney test; * p<0.05). Grouped dot plots showing fold change in expression for **(B)** CXCL10 and **(C)** IL-6. Resp., responders; Non-Resp., non-responders.

Plasma samples collected post-treatment (range 3–24 weeks after initiation of anti-PD1 treatment) were available for a subset of patients, including 15 responders (7 male, 8 female) and 6 non-responders (5 male, 1 female). A significant increase in the fold change expression of CXCL10 post-treatment was observed in non-responders (**Figure 3**). A 1.7 ± 0.6-fold change increase in CXCL10 expression was observed post-treatment in responders compared to a 2.4 ± 0.5-fold change increase (p<0.05) over baseline in non-responders to anti-PD1 therapy (**Figure 3**). A trend of increased IL-6 post-treatment in non-responders was also observed (p=0.057). Sample numbers were insufficient to analyze sex-related differences in post-treatment cytokine levels.

### 3.4 Plasma cytokines are predictive of PFS and OS

**Figure 4:**
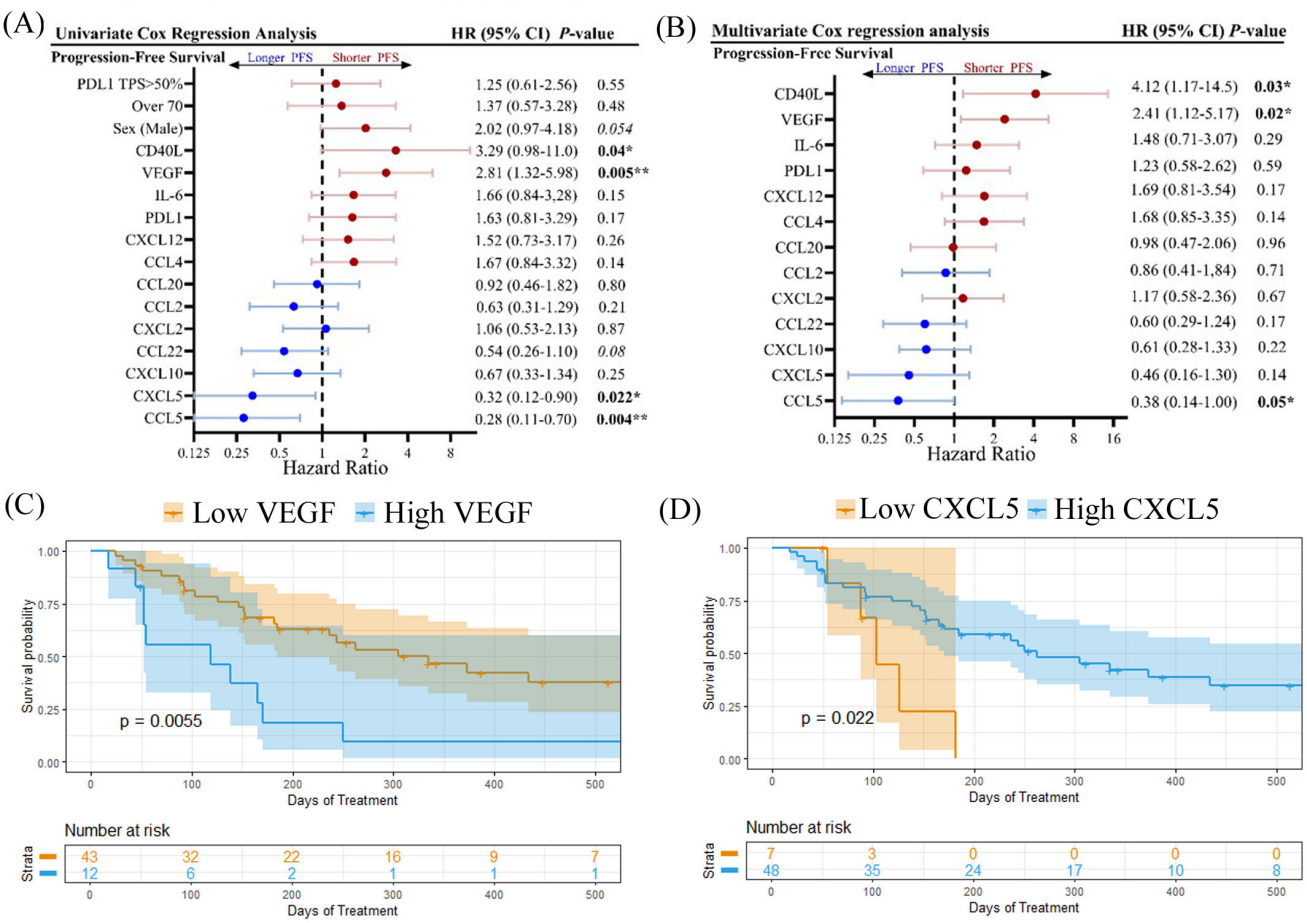
Forest plots showing **(A)** univariate Cox regression analysis and **(B)** multivariate cox regression analysis, adjusted for age and sex, for progression-free survival. Kaplan-meier plots showing percent progression-free survival for **(C)** VEGF and **(D)** CXCL5 are shown. Logrank p values are shown. *p<0.05, **p<0.01.

Univariate Cox proportional hazards model analysis was used to examine each cytokine for its correlation with PFS as a categorical variable based on a cut-off level determined by its capacity to discriminate between short and long PFS (**Figure 4A**). PDL1 TPS (>50%) and age (>70 years) were not found to be associated with shorter PFS, however as discussed previously, male patients experienced shorter OS compared to females (**Supplementary Figure S2A**). High levels of CD40L (HR=3.29; 95% CI 0.98-11.0; p=0.04) and VEGF (HR=2.81; 95% CI 1.32-5.98; p=0.005) were both found to be associated with shorter PFS. VEGF was also correlated to shorter OS (**Supplementary Figure S2**). Likewise, high VEGF levels were also associated with shorter OS (**Supplementary Figure S2**). Soluble CD40L and VEGF can both contribute to an immunosuppressive TME by expanding regulatory T cells (18, 19) and inhibiting migration and function of CD8+ T cells (20), respectively. In contrast, high levels of CXCL5 and CCL5 were found to be indicators of longer PFS (**Figure 4A**) and OS (**Supplementary Figure S2**). CCL5 has previously been identified as a marker of CD8+ T cell infiltration in NSCLC (21). Multivariate Cox proportional hazards model analysis (MVA) was also performed to adjust the data for sex and age (**Figure 4B**). High levels of CD40L and VEGF were negatively correlated to both PFS and OS (**Supplementary Figure S2B**) by MVA, while CCL5 was positively correlated to PFS but not OS. In addition, MVA revealed that high levels of IL-6 were negatively associated with OS (HR=3.03; 95% CI 1.00-9.16; p=0.05). IL-6 is a pro-inflammatory cytokine that can promote the immunosuppressive functions of myeloid-derived suppressor cells (MDSCs) (22). Kaplan-Meier plots showing the percentage of PFS (**Figures 4C, D**) and OS (**Supplementary Figure S1**) for VEGF and CXCL5 demonstrate that high baseline levels of these cytokines are associated with shorter PFS and OS.

**Figure 5:**
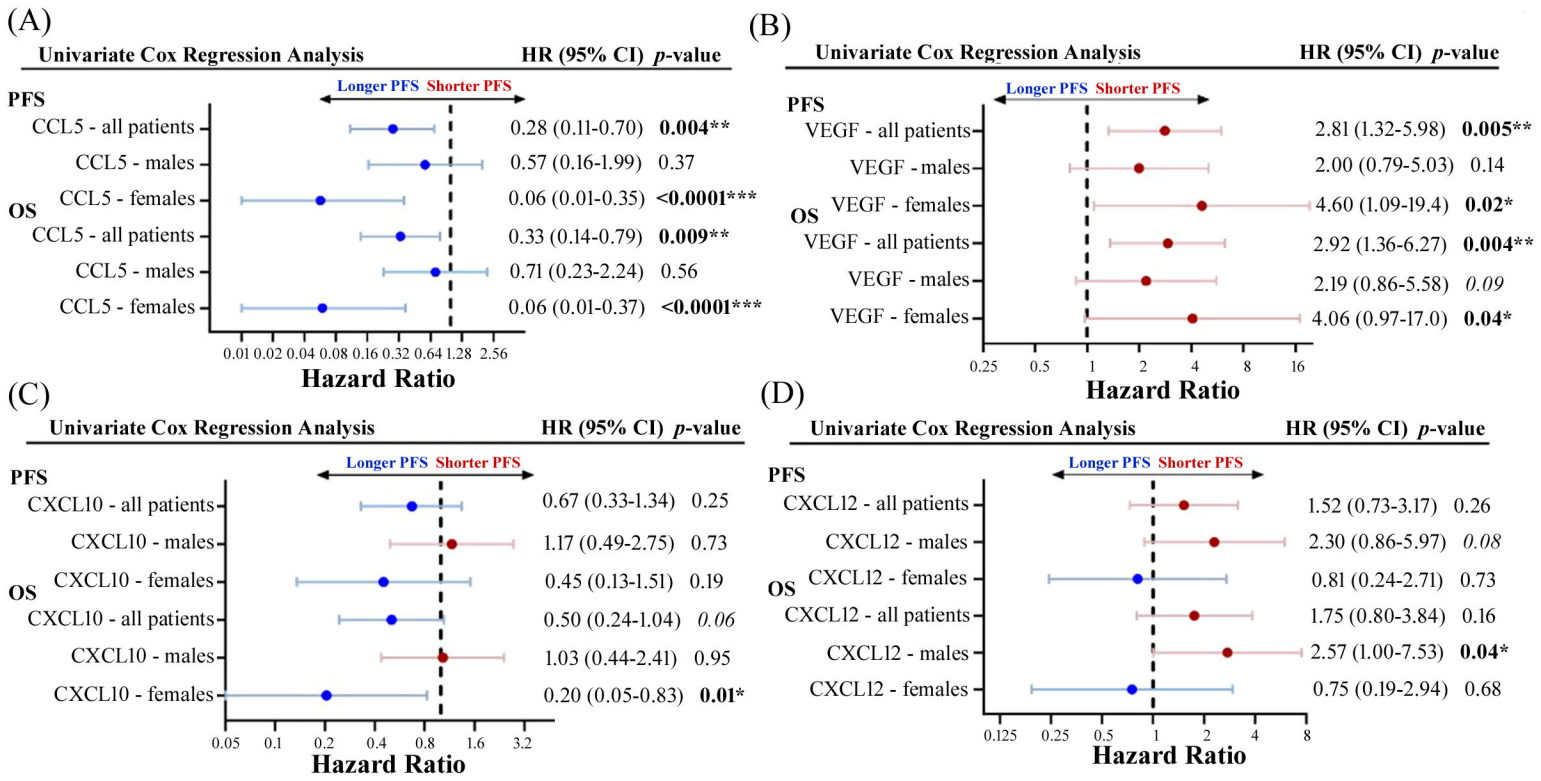
Forest plots showing univariate Cox proportional hazard analysis for males and females for both PFS and OS for **(A)** CCL5, **(B)** VEGF, **(C)** CXCL10, and **(D)** CXCL12. *p<0.05, **p<0.01, ***p<0.001.

**Figure 6:**
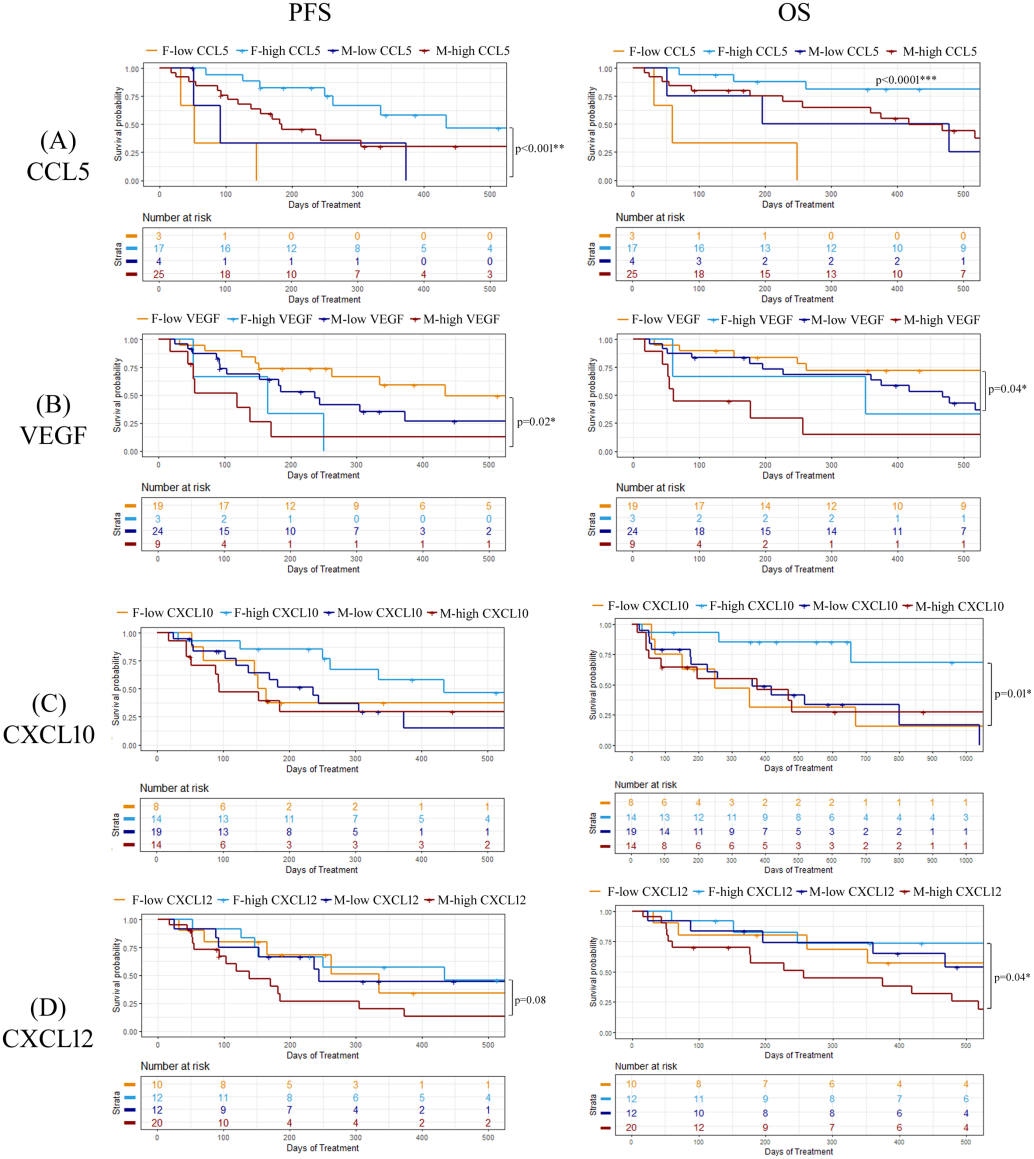
Kaplan-meier plots demonstration the percentage of PFS and OS for both male and female patients for **(A)** CCL5, **(B)** VEGF, **(C)** CXCL10, and **(D)** CXCL12. Logrank p values are shown. *p<0.05, **p<0.01.

To explore whether there were any sex-related differences between soluble cytokine biomarkers associated with PFS or OS, we performed univariate Cox proportional hazards model analysis independently on male and female patients. We identified four cytokines that had different associations with PFS or OS depending on sex, including CCL5, VEGF, CXCL10, and CXCL12 (**Figure 5**). As previously demonstrated, high levels of CCL5 were correlated to longer PFS and OS in the entire patient cohort, however when patients were separated by sex, CCL5 was found to only predict longer PFS and OS in female patients (**Figure 5A**). On the other hand, VEGF was found to be a predictor of shorter PFS and OS in female patients compared to males (**Figure 5B**). Similarly, CXCL10 was not found to have any predictive power of PFS or OS in male patients but was predictive of longer OS in female patients (**Figure 5C**). While CXCL12 was not found to be correlated to response, PFS, or OS when the entire patient cohort was analyzed, male patients with high CXCL12 were found to experience worse OS (HR=2.75; 95% CI 1.00-7.53; p=0.04) compared to male patients with low CXCL12 (**Figure 5D**). Furthermore, CXCL12 showed a trend towards a shorter PFS (HR=2.30; 95% CI 0.86-5.97; p=0.08) in male patients. In contrast, CXCL12 was not found to be predictive of either PFS or OS in female patients. These findings demonstrate the importance of including sex as an important determinant of response in biomarker studies. Survival curves for CCL5, VEGF, CXCL10 and CXCL12 segregated by sex are shown in **Figure 6**. Improved PFS and OS for female patients with high CCL5 compared to female patients with low CCL5 was observed (**Figures 6A, B**) as well as improved OS for females with high CXCL10 (**Figure 6F**) and worse PFS and OS for females with high levels of VEGF (**Figures 6C, D**). Shorter OS survival of males with high CXCL12 is also shown (**Figure 6H**). Independent survival curves for CCL5, VEGF, CXCL10, and CXCL12 for male and female patients are shown in **Supplementary Figure S3**.

### 3.5 UMAP analysis of cytokine clusters

**Figure 7:**
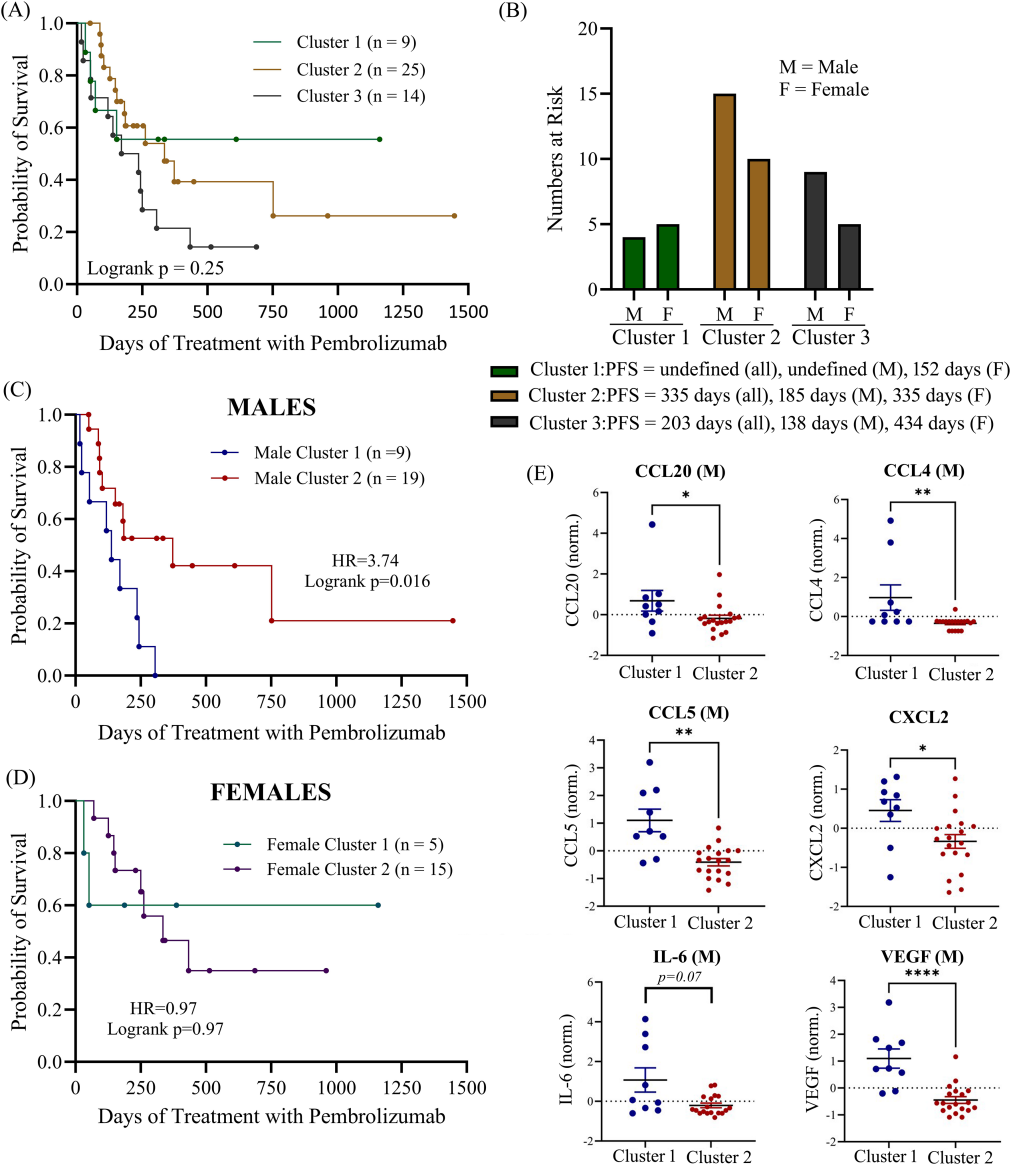
**(A)** UMAP of clustering of all patients in the cohort was used to identify 3 K-clusters of that were then correlated to progression-free survival. **(B)** number of male and female patients at risk in each cluster with median PFS indicated. Separate UMAP clustering (2 k-clusters) was performed for both males **(C)** and females **(D)**. **(E)** Normalized mean expression of most important principal components (CCL20, CCL4, CCL5, CXL2, IL-6 and VEGF) are shown for male patients. M, male; F, female; norm., normalized (normalized to reduce batch variability between assays). *p<0.05, **p<0.01, ****p<0.0001.

Since there are numerous mediators of immune regulation active in the TME which may act upon cytokines simultaneously, we decided to assess whether multiple soluble biomarkers in combination would be better predictors of ICI treatment response. We used UMAP to apply dimensional reduction to the cytokine dataset to identify patterns of cytokine expression that may be correlated to PFS following ICI treatment. UMAP is an approach which allows large datasets to be presented in a two- or three-dimensional manner while retaining the meaningful properties of high-dimensional data. First, we applied UMAP analysis to baseline cytokine values for the entire patient cohort and then used k-means clustering to divide patients having the most similar cytokine profiles into three separate clusters (**Supplementary Figure S4**). We did not observe any significant associations with PFS within the three different clusters (logrank p = 0.25; **Figure 7A**). To determine how sex may be impacting the results of the clustering, we identified the number of male and female patients in each of the three clusters and then calculated the median PFS for all the patients in the cluster as well as median PFS for males and females separately (**Figure 7B**). We found that males and females had a balanced distribution amongst the three clusters. However, in cluster 1, which had the longest median PFS (median PFS not reached), we found that the males in the cluster had an undefined median PFS while females in cluster 1 had a much shorter PFS of only 152 days. In contrast, in cluster 3, which had the shortest median PFS for all the patients in the cluster (203 days), we found that the males in the cluster had a very short PFS of only 138 days while the females in cluster 3 had a much longer PFS of 434 days. These findings suggest that even though these patients are clustering together based on their cytokine profiles, these clustering cytokines behave very differently as predictors of survival depending on the sex of the individual. Based on these findings, we then performed a separate UMAP analysis followed by K-means clustering, using only 2 clusters this time, on male and female patients individually to determine if we could find any clusters that were significantly associated with PFS (**Figures 7C, D**). Although we identified a cluster of male patients that was significantly associated with shorter PFS (“Male Cluster 1”), no clustering associated with PFS was observed in females (logrank p=0.97). The median PFS for males in “Male Cluster 1” was 138 days compared to 373 days for males in “Males Cluster 2” (HR=3.74; 95% CI 1.28-10.95; p = 0.016). Deconvolution of the UMAP data revealed that “Male Cluster 1” was characterized by higher levels of CCL20, CCL4, CCL5, CXCL2, IL-6 (near significance) and VEGF compared to individuals in “Male Cluster 2” (**Figure 7E**). These data suggest that there may be some interplay between these cytokines within the TME that is sex dependent. Pearson correlation analysis further highlighted this finding showing significant correlations between CCL20 with CCL4, IL-6 and VEGF, as well as correlations between CXCL2 and CCL5 (**Supplementary Figure S5**).

In summary, these findings indicate that baseline levels of cytokines, as well as on-treatment changes in cytokine levels with anti-PD1 therapy, can provide potentially useful prognostic biomarkers, particularly if male and female patients are analyzed separately rather than grouped together as is common practice is these types of studies.

## 4 Discussion

Despite accumulating evidence that sex is an important factor in influencing a patient’s immune responses as well as responses to drug therapy, sex is often overlooked in translational research studies as well as clinical decision making in the oncology setting (23). The existence of sex-related differences in the immune system are highlighted by the fact that females mount stronger innate and adaptive immune responses than males (6, 8, 9) and experience higher rates of autoimmune disorders (24), while males are more likely to succumb to malignant diseases and infections (25). Immune function is a dynamic process that changes during different life stages and reproductive status and is greatly impacted by signaling through sex steroid receptors. For instance, estrogen signaling can suppress natural killer (NK) cell function and increase Th1 responses and T cell tumor infiltration, while androgens can inhibit Th1 responses and reduce T cell anti-tumor immunity (26). Estrogen depletion has also been reported to alter the cytokine profiles and reduce macrophage polarization leading to reductions in tumor-infiltrating M2 macrophages in the liver TME (27). The influence of estrogen on lung cancer progression is evident by the fact pre-menopausal women are more likely to be diagnosed with more advanced, less differentiated lung cancer and have a worse prognosis than men or post-menopausal women (28). The presence of both high ERβ and aromatase expression is a negative prognostic of survival in lung cancer, particularly in post-menopausal women (12). Lung cancer cells also express androgen receptors which can act to increase estrogen’s pro-tumoral effects (11). Androgen receptors are over overexpressed in only ~ 20% of NSCLC patients and were associated with more advanced disease (29), while androgen deprivation therapy has been reported to improve survival in NSCLC if started after diagnosis (10).

NSCLC is typically a cancer that is diagnosed in an older population, and it is well established that the ability of the immune system to mount a robust defense against cancer declines with age, however this decline is not equivalent between the sexes (7). Due to age-related epigenetic changes, both sexes experience losses in T cell numbers, however older males experience a disproportionate decrease in T cell and B cell populations (30). It is only very recently that the importance an individual’s sex has on the efficacy of ICI has been recognized, due to the influence of sex chromosomes and sex hormones. Sex-related differences in response to immune checkpoint therapy have been reported but results of meta-analyses are not consistent (31). Although Conforti et al. (32) reported improved benefit of I-O therapy for females, a second meta-analysis found no such sex-related disparity in I-O response (33). These studies may be confounded by the inclusion of multiple cancer types in the analyses since better responses to anti-PD1 and anti-PDL1 treatments have been reported in females with NSCLC compared to males, however the opposite trend was observed in colorectal cancer (6). Furthermore, males have been reported to have better responses to ICI treatment on its own (34) while females had a significantly larger benefit when anti-PD1 therapy is combined with chemotherapy for treatment of lung cancer (14, 35). This may be in part a reflection of the finding that males tend to have tumors with a higher burden of mutations and antigenicity (36), while females tend to have more immune-infiltrated tumors (35) and thus benefit from the increase in antigens resulting from chemotherapy. Sex-related disparities in T-cell function required for adequate responses to anti-PD1 therapy in lung cancer have also been reported. For instance, higher levels of CXCL13, a cytokine correlated to T-cell tumor specificity, has been reported in T cells isolated from female compared to male patients (37). Although the patient number was small in this study, males were at significantly more risk of shorter OS than females in the study.

Sex hormones act as modulators of both pro- and anti-inflammatory cytokine production which contributes to differences in immune responses observed between the sexes, the effects of which depend both on hormone concentration (38) and the receptor subtypes expressed on target cells. Estrogen regulates cytokine expression mainly by binding specific nuclear receptors that bind to estrogen response elements (EREs) in the promoter region of target genes or via interaction with AP-1 and NF-kB transcription factors to regulate transcription (39). T cells express numerous cytokines whose promoters contain an ERE, including IFN-g, CX3CL1, IL-1, and IL-16 (40). Furthermore, estrogen has been reported to increase CD4+ T cell expression of CC chemokine receptors 1-5, which may explain the increased sensitivity of the T cell chemokine receptor response and T cell homing in females (41). The enhanced T cell responsiveness to CCL5 observed in females may have contributed to the improved outcomes observed in females with high CCL5 plasma levels in our study.

Elevated baseline levels of circulating CD40L were associated with shorter PFS and OS in this study. While CD40L, which mediates interactions between T cells and B cells, is anti-tumoral, the soluble form of CD40L that circulates in the blood has an immune-suppressive effect in the TME. Soluble CD40L can promote tumor progression by increasing MDSCs and Tregs in the TME and inhibiting T cell expansion (18–20) and may be a negative prognostic of survival in NSCLC (42).A few recent studies have reported sex-related differences in circulating cytokines in patients treated with immune checkpoint therapy. In 2024, Pasello et al. (43) reported that higher baseline levels of IL-4, sPDL1, and IL-10 in females and IL-6 and VEGF in males was associated with an increased risk of progression. Passelo et al. also reported that IL-6 and VEGF were significantly lower at baseline in patients, both male and female, who experienced either a complete or partial response, compared to patients with stable or progressive disease. This is similar to results reported here demonstrating that higher levels of IL-6 and VEGF were associated with reduced OS. IL-6 has previously been identified as a poor prognostic for survival in NSCLC patients treated with ICI (16, 17)) and may contribute to an immune-suppressed TME through its regulation of MDSCs (22). However, in our study we also identified VEGF as a better predictor of shorter PFS in females compared to males. Estrogen also influences angiogenesis through the regulation of VEGF and its receptor, VEGFR2, particularly in reproductive tissues but also in ER-α positive tumors where estrogen can promote tumor growth and metastasis via increased vascularization (44). The estrogen-mediated upregulation of VEGFR2 in tumors may enhance VEGF responsiveness and increase angiogenesis. Estrogen is known to increase angiogenic factors, including VEGF, and estrogen has been reported to promote myeloid recruitment and resistance to VEGF-targeted therapies (45). Therefore, sex-related differences in VEGF production and function may help explain the observation in this study that VEGF in females was a stronger predictor of short PFS than in males.

A number of studies have identified CXCL10 as a negative prognostic of PFS in NSCLC patients at baseline (15). CXCL10 is a secreted chemokine that is involved in trafficking of CXCR3-positive leukocytes, including CXCR3+ tumor associated CD8+ T cells and natural killer cells that promote tumor suppression (46) as well as immunosuppressive CXCR3+ Tregs (47). Thus, CXCL10 exerts both pro- and anti-tumoral effects in the TME and the effects may be dependent on the type of cancer. For example, high CXCL10 levels were associated with CD4+ and CD8+ T cell infiltration in the TME in breast cancer (48), but in CRC and NSCLC high CXCL10 levels were associated with shorter survival (15, 49). In this study, we find that the opposing effects of CXCL10 may be further complicated by sex-related differences in response to high levels of CXCL10, whereby high CXCL10 levels were found to be associated with longer OS only in female patients. CXCL10 levels have been reported to be higher in females (50) due to regulation by type 1 interferons (51) and x-linked TLR7 expression (52). Furthermore, androgen blockade has been demonstrated to increase CXCL10 and associated CD8+ lymphocyte infiltration, suggesting that androgens may suppress CXCL10 and T cell recruitment (53, 54). Although we did not have sufficient numbers of patients with post-treatment samples to attempt to analyze sex-related differences, we did observe, as previously reported in lung cancer patients (16), a significant increase in CXCL10 in non-responders while on-treatment, as well as a near-significant increase in IL-6. Elevated levels of CXCL10 following anti-PD1 treatment was observed in melanoma patients who responded to immune checkpoint blockade (55). However, in concordance to the findings of this study, increased on-treatment CXCL10 was associated with poor PFS in lung cancer patients treated with anti-PD1 (16).

CXCL5 is a known inducer of neutrophil infiltration and has been associated with neutrophil proportion and negative prognosis in many studies (56). In this study, we identified CXCL5 as being positively associated with PFS and OS when univariate Cox proportion hazards model was performed on the entire patient cohort but not when males and females were analyzed separately. Although no differences in CXCL5 plasma levels were observed between males and females or between responders and non-responders when the whole cohort was analyzed, we did observe a significantly higher level of CXCL5 in male responders compared to male non-responders. Sex disparities in induction of CXCL5 has been observed in rodent reperfusion models and CXCL5-driven neutrophil recruitment was observed only in males in response to pro-inflammatory stimuli (57). Furthermore, CXCL5 has been reported to be influenced by androgen signaling (58). The effect of CXCL5 may depend on the source since it can be secreted by both cancer cells or cells in the TME such as macrophages and dendritic cells (59).

A novel finding of this study was the association of high levels of baseline CXCL12 with poor disease control in male but not female patients. CXCL12 acts through the C-X-C motif receptor 4 (CXCR4) to promote the recruitment of CXCR4+ neutrophils and leukocytes to the TME (60) as well as to facilitate metastasis of lung cancer (61). Although high baseline serum CXCL12 levels have previously been reported to be correlated to shorter PFS and OS (62), no sex-based analysis was performed. Sex-related differences in the regulation of CXCL12/CXCR4 signaling have been reported. Mouse models have demonstrated that male mice exposed to chlorine gas have higher rates of lung injury and mortality than female mice due to higher levels of CXCL12-CXCR4 signaling in males leading to enhanced tissue migration of neutrophils and leukocytes (63). The chemokine CXCL12 along with its receptor, CXCR4, have been implicated in the progression and metastasis of cancers, including lung cancer (60). Estrogen has been demonstrated to increase CXCL12 expression by ER-positive tumors (64), including lung cancer (65), leading to proliferation and enhanced invasiveness of the tumor. Male sex hormones, including testosterone, have also been reported to influence CXCL12 production (66) and correlation between AR and CXCL12 expression was observed in breast cancer tissue (67). Although it is clear that sex hormones influence production of CXCL12 and its cognate receptor CXCR4, it is unclear why we observe sex-related difference on the impact of increased CXCL12 on survival in this study. It could be due to differences in the cell population of TME and their expression of appropriate receptors, changes in sensitivity to CXCL12/CXCR4 signaling due to smoking history, genetic differences, or interactions between sex hormones. In this study, we found that elevated CXCL12 was only a negative prognostic for males. Androgen-mediated up-regulation of HIF-1a and CXCL12 was observed in male but not female endothelial cells (68), suggesting the possibility that male-specific effects of CXCL12 on survival could be in part due to increased angiogenesis. There is a great deal of interest in the use of CXCR4 antagonists to improve ICI therapeutic benefit, which has shown promise in *in vitro* studies (69, 70). Our findings reported here that CXCL12 is a better prognostic indicator of poor response to ICI therapy in males, as well as reports that CXCL12 and CXCR4 expression is higher in males (63, 71) suggests that response to CXCR4 inhibition may be sex dependent.

Although circulating baseline levels of CCL4, CCL20, and CXCL2 were not predictive of survival when analyzed individually, UMAP and K-means clustering analysis identified a subgroup of male patients with short PFS that were characterized by high baseline levels of VEGF, IL-6, CCL4, CCL20, CXCL2, and CCL5. CCL20 is that contributes to tumor evasion by recruiting Tregs and Th17 cells to the tumor niche, promoting EMT transition and is a negative prognostic for immunotherapy treatment (72). CCL4 can have both anti- and pro-tumoral effects through its recruitment of immune cells, in particular CD8+ T cells, MDSCs and Tregs, to the TME (72). CXCL2 also plays a critical role in immune infiltration and modulation of the TME via its interaction with tumor-associated macrophages (TAMs) and neutrophils (73). This sex-specific clustering of chemokines may reflect the differences reported in the type of immune cells within the TME that has been observed between males and females (38, 40, 41, 53, 74). The male TME is more often enriched in immune-suppressive cells such as tumor-associated macrophages, Tregs and MDSCs which secrete anti-inflammatory cytokines, such as IL-6, and chemokines that lead to reduced T cell function (74). These data further suggest that sex may influence the interplay of cytokines within the TME.

The role of sex hormones in the field of immunotherapy is an important consideration since both estrogens, progesterone, and androgens can facilitate immune evasion by modifying immune checkpoint pathways (13, 74), modulating immune cells within the TME, and facilitating CD8+ T cell exhaustion (75). Although estrogen can promote activation of immune cells, estrogen signaling within the TME promotes an immunosuppressive environment via suppression of CD8+ T cells and support of myeloid derived suppressor cells (76). Furthermore, in ER-positive breast cancer, hormone therapy in combination with anti-PD1 immunotherapy increased immune cell activation and infiltration into the TME, suggesting blocking estrogen activity may improve immunotherapy responses (77). Importantly, disruption of estrogen signaling may also improve the anti-tumoral activity of immunotherapy even in hormone-independent metastatic cancers (27). Since testosterone has more immunosuppressive activity compared to estrogen (78), males with higher testosterone may have less robust anti-tumoral responses with anti-PD1 therapy. Furthermore, androgen deprivation therapy increased the infiltration of CD8+ T cells into the TME, suggesting that blocking androgen signaling may improve immunotherapy responses (53, 54).

Overall, very few studies have considered sex as a variable when analyzing these types of studies, despite the well documented differences in immunity and response to ICIs between the sexes. This may be in part due to the increased incidence of NSCLC in males that leads to studies that are heavily weighted towards males (23, 56). Future studies would benefit from additional cohort information with respect to sex hormone replacement therapy status, peri-menopausal or post-menopausal status, castration status, the presence of sex-specific tumor mutations, and gender affirmation therapy. In conclusion, the findings of this study highlight the importance of considering sex when interpreting findings from immune-oncology precision medicine research studies to improve accuracy of predictive biomarkers and facilitate better standard of care and treatment outcomes.

## Data Availability

The raw data supporting the conclusions of this article will be made available by the authors, without undue reservation.
