# Supplementary material for: Sex-specific cytokine signatures as predictors of anti-PD1 therapy response in non-small cell lung cancer
Source: Front Immunol. 2025 Jun 16;16:1583421. doi: 10.3389/fimmu.2025.1583421 (PMC12206799; doi:10.3389/fimmu.2025.1583421)

## Supplemental Figures

Supplemental Figure S1: Kaplan-meier plots demonstration the percentage of overall survival for KM plots for Overall survival for A) Sex, B) CCL5, C) CXCL5, and D) VEGF. Logrank p values are shown.

(A)

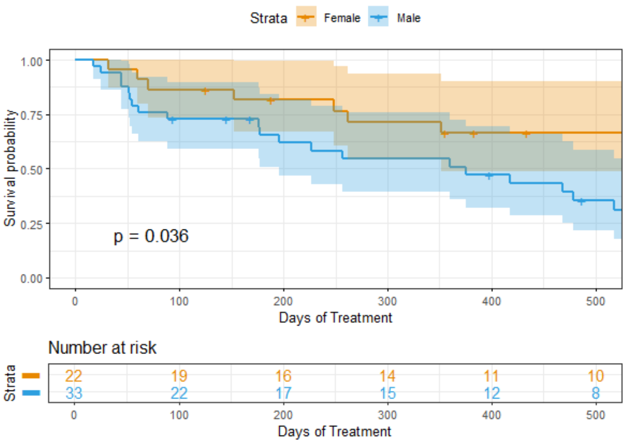


(B)

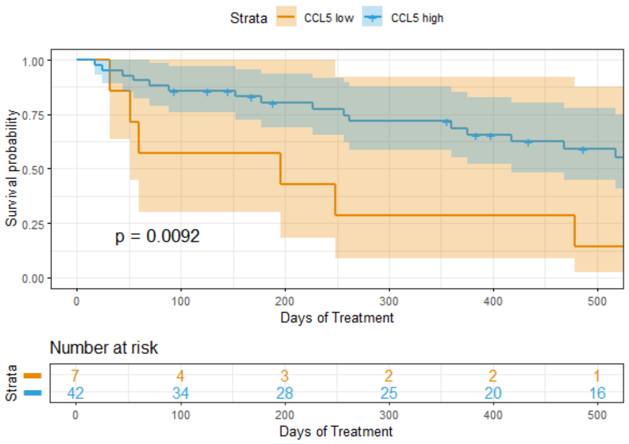


(C)

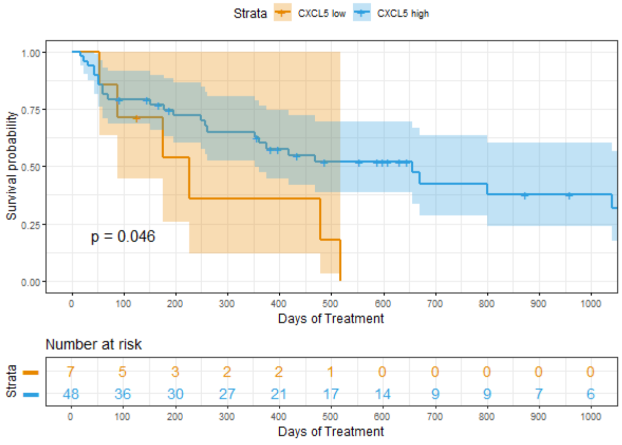


(D)

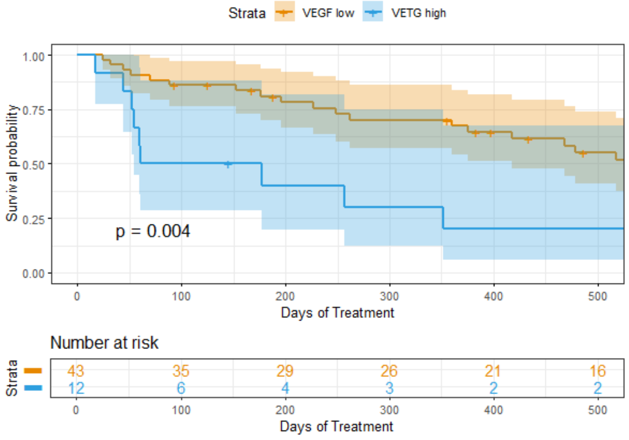

Supplemental Figure S2: Forest plots showing (A) univariate or (B) multivariate Cox proportional hazard analysis for overall survival.

(A)

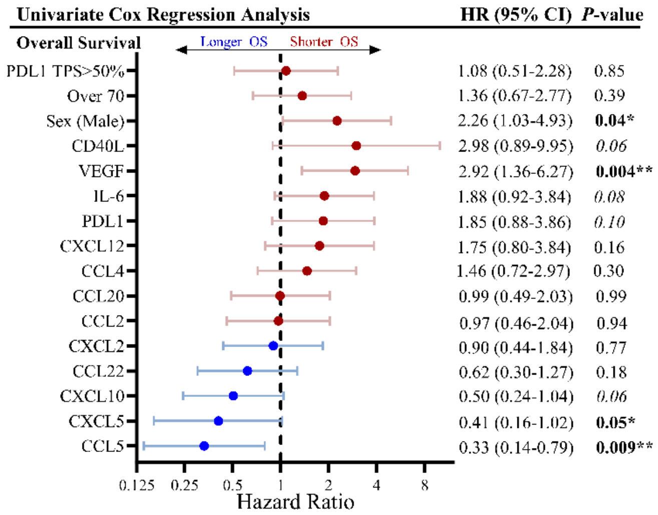


(B)

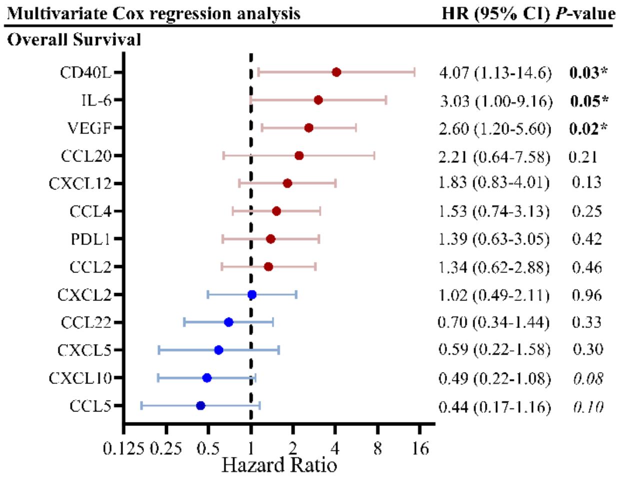

Supplemental Figure S3: Kaplan-meier plots for overall survival for male and female patients for A) CCL5, B) VEGF, C) CXCL10 and D) CXCL12. Logrank p values are shown.

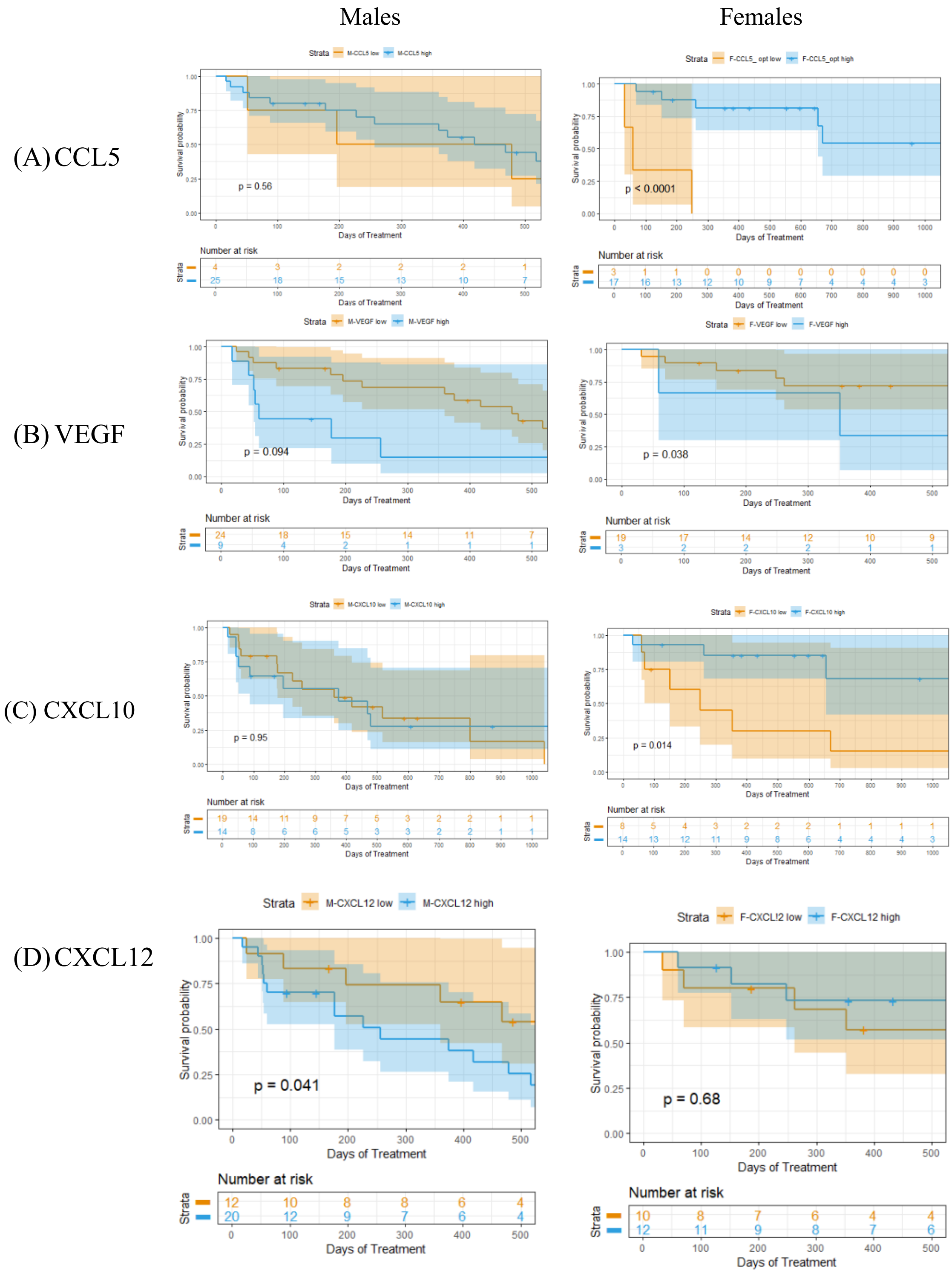

Supplemental Figure S4: UMAP of all patients using 3 k-clusters with responder, non-responder and non-evaluable patients indicated.

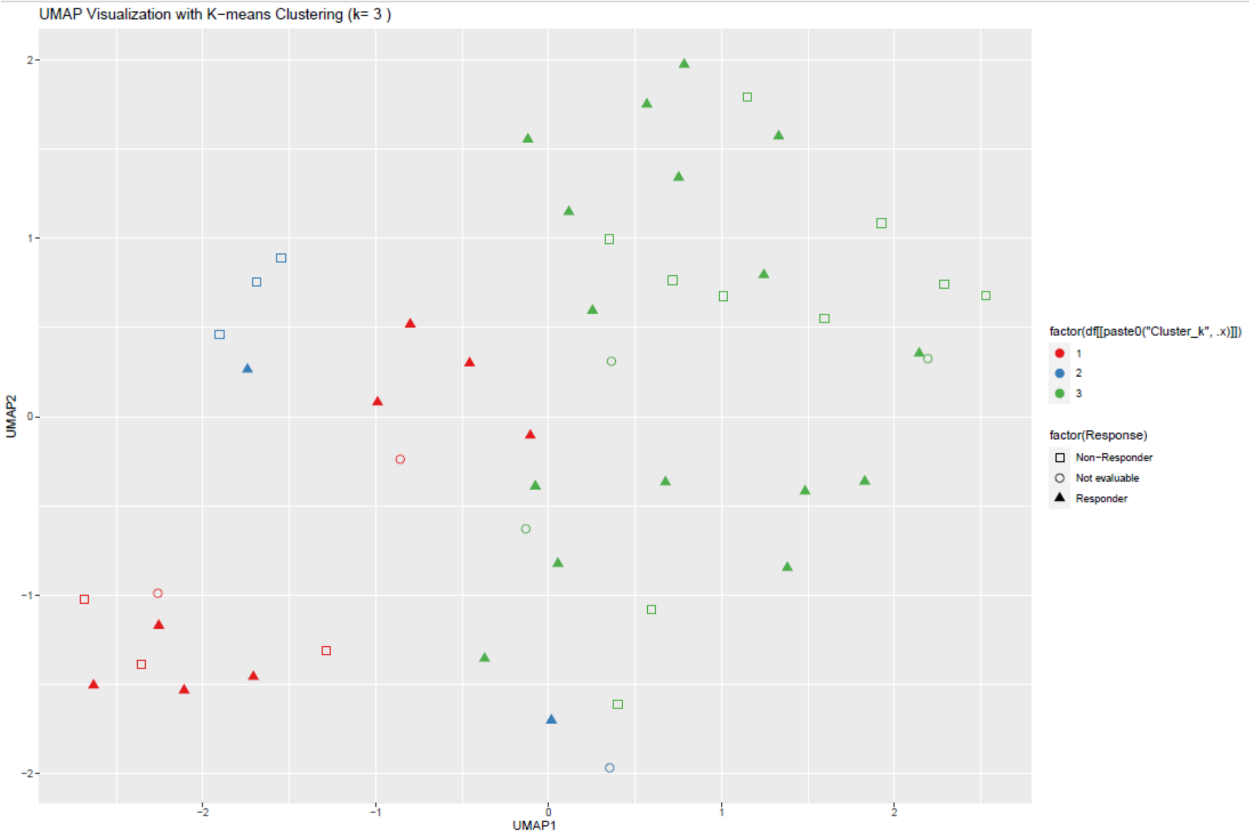

Supplemental Figure S5: Pearson Correlation Matrix. All circles indicate significance levels ($p<0.05$), with larger circles indicating greater significance levels.

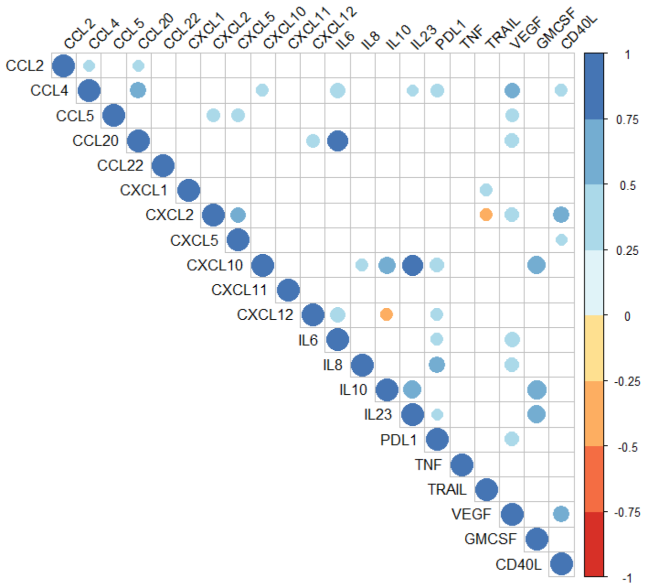

Supplemental Figure 6: UMAP of A) male patients and B) female patients with 2-k clusters and responder, non-responder and non-evaluable patients indicated

Males (PC4/k2)

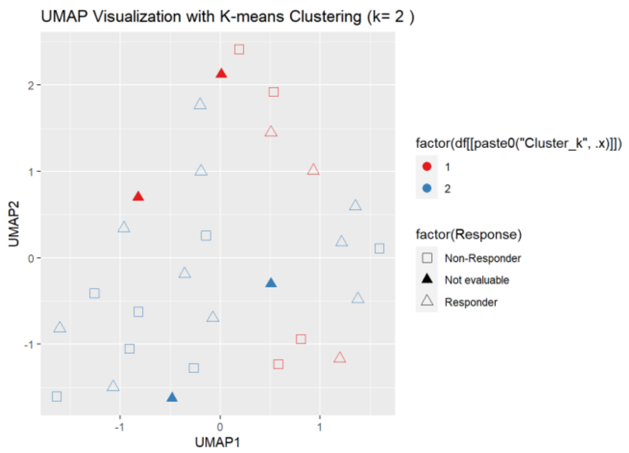


Females (PC4/k2)

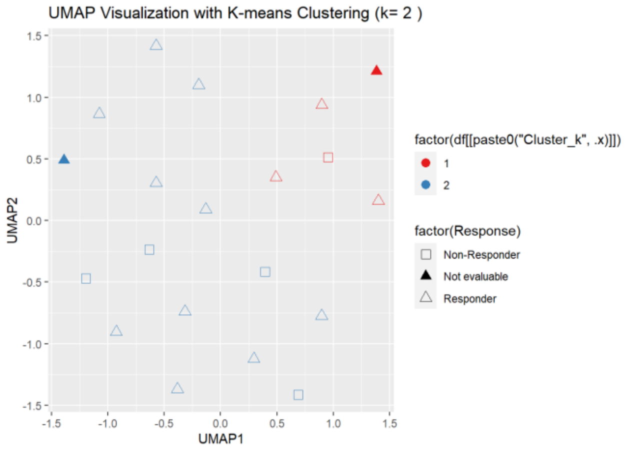

Supplement: Supplementary file 1 [file DataSheet1.pdf]
